# Increased rod stiffness improves the degree of deformity correction by segmental pedicle screw fixation in adolescent idiopathic scoliosis

**DOI:** 10.1186/1748-7161-6-13

**Published:** 2011-07-28

**Authors:** Kasim Abul-Kasim, Magnus K Karlsson, Acke Ohlin

**Affiliations:** 1 Faculty of Medicine, Lund University, Division of Neuroradiology, Diagnostic Centre for Imaging and Functional Medicine, Skåne University Hospital, 205 02 Malmö, Sweden; 2 Faculty of Medicine, Lund University, Department of Orthopaedic Surgery, Skåne University Hospital, 205 02 Malmö, Sweden

## Abstract

**Background:**

There are limited reports in literature studying the impact of rod diameter and stiffness on the degree of deformity correction in patients with AIS.

**Aims:**

The aims of this study were to evaluate the 3-dimentional deformity correction achieved by segmental pedicle screw fixation in patients with adolescent idiopathic scoliosis, and to find out if learning or the change to stiffer rods had any positive impact on deformity correction.

**Study design:**

Retrospective study.

**Methods:**

Plain radiographs and low-dose spine CTs of 116 consecutive patients (aged 15.9 ± 2.8 years) operated during the period 2005-2009 (group 1: patients operated autumn 2005-2006; group 2: 2007; group 3: 2008; group 4: 2009) were retrospectively evaluated.

**Results:**

There was no statistically significant difference between the correction of the Cobb angle (P = 0.425) or lower end vertebra tilt (P = 0.298) in patients operated during the first versus the remaining periods of the study. No restoration of the sagittal kyphosis was reported in the first period compared with 5.9° in the last study period (P < 0.001). The correction of vertebral rotation was also improved from 4.2° to 7.8° (P < 0.001) for the same periods. For the whole study population, there was statistically significant correlation between the order of the operation (patient number) and the restoration of sagittal kyphosis (r = -0.344, P = 0.001), and the correction of vertebral rotation (r = 0.370, P < 0.001), but not for the Cobb angle or LEVT. However, there was no significant difference in restoration of sagittal kyphosis and the vertebral rotation in the first 17 patients compared with the last 17 patients operated with rods of 5.5 mm diameter (P = 0.621, and 0.941, respectively), indicating that rod stiffness had more impact on the deformity correction than did learning.

**Conclusions:**

This study showed that rod stiffness had more impact on the deformity correction than did learning.

## Background

The method of using segmental pedicle screw fixation in scoliosis surgery was presented by Suk in 1994 [1]. Recently a quantitative study showed that rod derotation and direct vertebral derotation can significantly improve the 3-dimensional correction of scoliotic deformity [2]. Besides lateral curvature of vertebral column in the coronal plane, scoliotic deformities also include changes in the sagittal plane such as thoracic hypokyphosis or even lordosis and are always associated with rotation of vertebral bodies in the axial plane. While standing plain radiography still is the most common method to assess the correction in coronal and sagittal planes, studies have shown that computed tomography (CT) is superior to plain radiography in the assessment of the degree of vertebral rotation [3,4]. Low-dose spine CT with significant reduction of the radiation dose has recently been introduced as a reliable method in the work-up of scoliosis [5]. To the best of our knowledge there is no report on assessment of the degree of vertebral derotation after scoliosis surgery using low-dose spine CT. Furthermore, the published reports on the correction of vertebral rotation often present the correction rates for the whole study period, which probably overshadow the improvement of correction ability over time-"the learning curve" for the surgeon. The knowledge on the effect of learning and cumulative experience has, to our knowledge, not previously been studied with regard to the deformity correction in all three planes by using the most reliable methods for evaluation of deformity in each plane.

The Suk technique was introduced at the Orthopaedic Department of our hospital in autumn 2005, after a study visit to Seoul by the senior author. The implants used were made of titanium alloy with rod diameter of 5.5 mm during 2005-2006; thereafter, the rod diameter was 6.35 mm. The change in radius alters stiffness to the 4^th ^power of the change in radius. Consequently, the bending stiffness has increased from 5.17 EI (Nm^2^) for rod diameter of 5.5 to 9.18 EI (Nm^2^) for rod diameter of 6.35 mm. The stiffness of a rod is related to its ability to hold correction and minimize changes in rod curvature; the stiffer the rod, the less changes [6]. Previous studies have shown that greater construct stiffness was at least partially dependant on the diameter of rod of the constructs [7,8]. However, the anchors used in these 2 experimental studies were not pedicle screws.

The primary aim of this study was to evaluate the 3-dimentional deformity correction achieved by posterior corrective surgery using "all pedicle screw construct" in patients with adolescent idiopathic scoliosis (AIS). The second aim of the study was to find out if there was any improvement in the degree of deformity correction during different periods of the study and whether this improvement was dependant on learning or the change to rods of larger diameter.

## Methods

Pre- and postoperative plain radiographs and low-dose spine CTs of 116 consecutive patients with AIS who underwent posterior corrective surgery with titanium-alloy (Ti-6AI-4V) during the period 2005-2009 were evaluated retrospectively. The patients were categorized into four period groups according to the date of operation (group 1: patients operated on in autumn 2005-2006, group 2: 2007, group 3: 2008, and group 4: 2009). As the number of patients operated on in autumn 2005 was only 7, these were included in the first period with patients operated on in 2006. Ninety four patients (81%) were female and 22 patients (19%) were male. The patients were identified from the database of the Orthopaedic Department and the database of the Radiology Department of our hospital. All patients had been examined with standing posteroanterior (PA) and lateral radiographs as well as with low-dose spine CT before surgery and six weeks postoperatively. The measurements performed on plain radiographs before and after surgery were: (1) Cobb angle of the major curve on standing PA-radiographs, (2) The degree of kyphosis at T5-T12 on standing lateral radiographs, and (3) Lower end vertebra tilt (LEVT), which is defined as the angle between the lower endplate of the lower end vertebra of the major curve and the horizontal plane, measured on standing PA-radiographs.

All CT-examinations were performed on a 16-slice scanner (SOMATOM Sensation 16, Siemens AG, Forchheim, Germany) according to our low-dose spine CT protocol: Slice collimation 16 × 0.75 mm, rotation time 0.75 s, pitch 1.5, tube voltage 80 kV and quality reference for the effective tube current-time product 25 mAs. The measurement of vertebral rotation was performed at the most rotated vertebra at the apex of the major curve as well as at one adjacent vertebra on either side of the apex. These measurements were performed at the same vertebral levels before and after surgery. The degree of vertebral rotation was measured according to the method of Aaro and Dahlborn [9]. All measurements were performed by an experienced radiologist with specialized spine profile. The pre- and postoperative data which were collected from the medical and the radiological records of the patients included: age, gender, diagnosis, Lenke classification, date of operation, and the type of the operation.

The approval of the Regional Radiation Protection Committee to use the low-dose spine CT in the work-up of patients with AIS was obtained.

### Operative technique

All operations were performed under general anesthesia with spinal cord monitoring using motor evoked potentials (MEP). The operations were performed through a standard posterior midline incision. After exposure of the posterior bony elements, estimated entry points for screws were defined by means of anatomical landmarks and small holes were created with an awl. Titanium markers were introduced into the holes with an estimated trajectory. Prior to making the screw canal, a fluoroscopic check of the accuracy of pin position and direction was made for each pedicle. The first part of the screw canal was made by means of a hand drill with a diameter of 3.2 mm, followed by a thin probe or feeler. The patency of the screw canal was checked with a feeler probe. Self-tapping screws with uniplanar screw head construct was regularly used. Pedicle screws used in this study were exclusively uniplanar. Curve correction was performed with a simple rod derotation manoeuvre of 90° of the concave rod. A firm pressure was applied to the hump on the convexity as well as applying pressure on direct vertebral rotation (DVR) handles attached to the heads of the 3-4 apical screws at the convexity, to counteract an increased vertebral rotation which otherwise often occur during the simple rod derotation. Before introduction of the stabilising rod on the convexity, DVR [10] was performed, in the apical area as well as in the caudal part of the construct if a significant rotation existed. As an attempt to improve the degree of deformity correction in stiffer curves, in situ concave rod bending and a more aggressive soft tissue release were performed when considered necessary. Prior to attachment of the convex rod, destruction of intervertebral joints as well as decortication of bony surfaces were performed.

### Statistical analysis

All statistical analyses were performed by means of SPSS version 17. Data is presented as proportions (%) or as mean ± with standard deviations (SD) or with 95% confidence interval (95% CI). A linear regression analysis was performed to test for the occurrence of a trend of successively improved skill to correct the spinal deformity in all three planes during the study period 2005-2009. Pearson correlation was tested between patient number (order of the operation) and the degree of deformity correction. Independent sample t-tests were performed to test for the significance of differences in deformity correction between different study periods. Patients operated on with a rod diameter of 5.5 mm during 2005-2006 (n = 34), all were performed by or under supervision of the senior surgeon (AO), were divided into two groups (the first 17 and the last 17 patients) thereafter independent sample t-tests were performed to compare the magnitude of deformity correction among patients of the two groups. Spearman correlation and Mann Whitney-U tests were used to test the association between the correction of deformity and different variables such as age, gender and curve type. The statistical significance was set to P < 0.05.

## Results

### Patient and curve characteristics

Out of the 116 included patients, 34 operated during 2005-2006, 21 during 2007, 25 during 2008, and 36 during 2009, with mean age of 15.9 ± 2.8 years, median 15 years and range 12-24 years. The types of scoliotic curves according to Lenke classification [11] was 60 Lenke type 1 (52%), 7 Lenke type 2 (6%), 19 Lenke type 3 (16%), 5 Lenke type 4 (4%), 15 Lenke type 5 (13%), and 10 Lenke type 6 (9%). The Lenke curve types were evenly distributed among patients of the different study groups. The major structural curve was thoracic in 90 patients (78%) and lumbar in 26 (22%). No vascular or infectious complications were recorded after surgery. One patient reported postoperatively pain and paraesthesia in the T8-T10 dermatome. The neurological deficit was believed to be caused by local extraforaminal nerve injury as low-dose spine CT showed no screw misplacement.

### Deformity correction for the whole study cohort

The mean reduction of Cobb angle was 38.4 ± 8.9° (69%), LEVT 19 ± 6.9° (68%), and vertebral rotation 6.5 ± 3.9° (37%) (all P < 0.001, respectively). The mean restoration of thoracic kyphosis was 4 ± 5° (24%) (P < 0.001), Table 1.

**Table 1:** The results of the linear regression analyses of the deformity correction during the study period (2005-2009)

	Preoperative	Postoperative	Correction (%)	Coefficient (95% CI)	P-value
**Cobb angle:**					
Whole study cohort	55.8 ± 10.2	17.3 ± 7.7	38.4 (69)		
Period 1	57.3 ± 8.6	19.9 ± 7.2	37.4 (66)		
Period 2	59.4 ± 13.7	21.2 ± 8.4	38.2 (64)		
Period 3	54.6 ± 8.9	16.1 ± 9.5	38.5 (72)		
Period 4	53.2 ± 9.6	13.6 ± 3.9	39.6 (74)		
				0.7 (-0.7/2.1)	0.325

**LEVT:**					
Whole study cohort	27.8 ± 6.7	8.9 ± 5.3	19 (68)		
Period 1	27.1 ± 6.3	9.2 ± 4.5	17.9 (66)		
Period 2	29.3 ± 9.4	10.4 ± 6	18.9 (64)		
Period 3	28 ± 5.3	9 ± 6.5	19 (68)		
Period 4	27.5 ± 6.2	7.6 ± 4.3	19.9 (71)		
				0.6 (-0.4/1.7)	0.237

**Sagittal profile:**			Kyphosis restoration		
Whole study cohort	15.1 ± 8.1	19.1 ± 6.3	4 (24)		
Period 1	16.6 ± 9.1	16.2 ± 8.3	-0.4 (2)		
Period 2	15 ± 6.9	19.8 ± 6.1	4.8 (32)		
Period 3	14.6 ± 9.1	19.9 ± 5.8	5.3 (36)		
Period 4	14.2 ± 7.7	20.1 ± 4.6	5.9 (41)		
				1.9 (0.9/3)	< 0.001

**Vertebral rotation:**					
Whole study cohort	18.4 ± 6.4	11.9 ± 6.2	6.5 (37)		
Period 1	16.6 ± 5.7	12.4 ± 6.2	4.2 (29)		
Period 2	18.9 ± 6.8	12.4 ± 5.9	6.5 (36)		
Period 3	19.8 ± 6.4	12.3 ± 5.7	7.5 (39)		
Period 4	18.8 ± 6.6	11 ± 6.3	7.8 (45)		
				1.2 (0.6/1.8)	< 0.001

LEVT indicates lower end vertebra tilt.

(95% CI) indicates 95% confidence interval.

Preoperative, postoperative, correction, and coefficient and the 95% CI values are given in degrees

Kyphosis restoration is the product of the postoperative sagittal Cobb-preoperative sagittal Cobb.

### Deformity correction during different study periods

**Figure 1:**
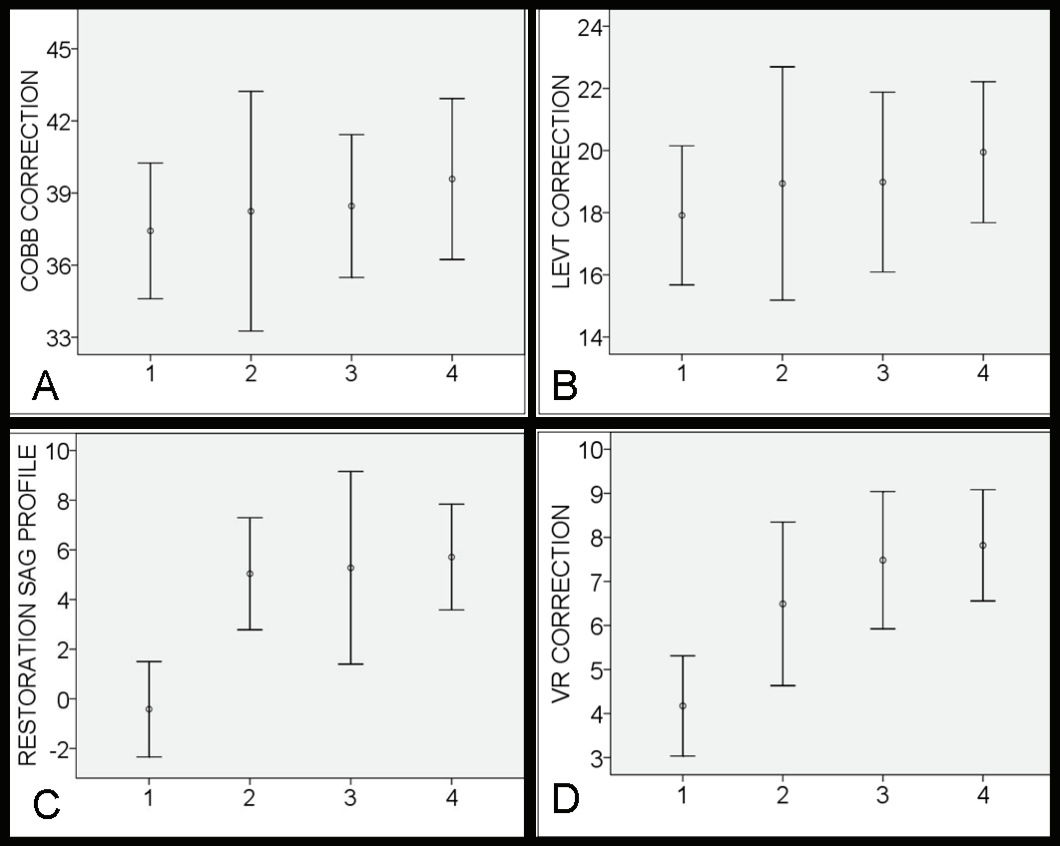
**Error bars showing the mean value and the 95% CI (give in degrees) of the correction rate of Cobb angle (A), LEVT (B), restoration of the sagittal profile (C), and vertebral rotation (D) against the four different study periods**. Patients operated on in autumn 2005 and in 2006 referred to as period 1, 2007 as period 2, 2008 as period 3, and 2009 as period 4. Footnotes: LEVT indicates lower end vertebra tilt, and VR indicates vertebral rotation.

The linear regression showed that the correction of the deformities in coronal plane improved from 37.4° (66%) in 2005-2006 to 39.6° (74%) in 2009 (P = 0.325), Figure 1A, and Table 1, and the correction of LEVT from 17.9 ° (66%) in 2005-2006 to 19.9° (71%) in 2009, (P = 0.237), Figure 1B, and Table 1. The measurement of the sagittal Cobb angle showed no restoration of thoracic kyphosis in 2005-2006 compared to restoration of kyphosis of 5.9° in 2009, (P < 0.001), Figure 1C, and Table 1. The correction of apical vertebral rotation was improved from 4.2° (29%) in 2005-2006 to 7.8° (45%) in 2009, (P < 0.001), Figure 1D, and Table 1.

On group level, there was no significant difference between the correction of the Cobb angle (P = 0.425), and LEVT (P = 0.298) in patients operated on during the first period (2005-2006) compared with that in patients operated on during the remaining periods (2007-2009). In contrast, the mean restoration of the sagittal profile (P < 0.001) and the mean correction of the vertebral rotation (P < 0.001) was significantly better in patients operated 2007-2009 than in patients operated 2005-2006, Table 2.

**Table 2:** The results of the independent sample t-test to find out if there were break points (significant differences in the deformity correction) between the first period i.e. at the end of 2006 compared with 2007-2009

Difference between the pre- and the postoperative measures of:	Mean difference period 1 against period 2-4	95% CI		P-value
		**Lower bound**	**Upper bound**	
			
Cobb angle	1.5°	-5.1°	2.2°	0.425
LEVT	1.5°	-4.3°	1.3°	0.298
Restoration of thoracic kyphosis	5.9°	3°	8.7°	< 0.001
Vertebral rotation	3.2°	4.7°	1.7°	< 0.001

LEVT indicates lower end vertebra tilt.

95% CI indicates 95% confidence interval.

### Correlation between the deformity correction and the order of the operation

**Figure 2:**
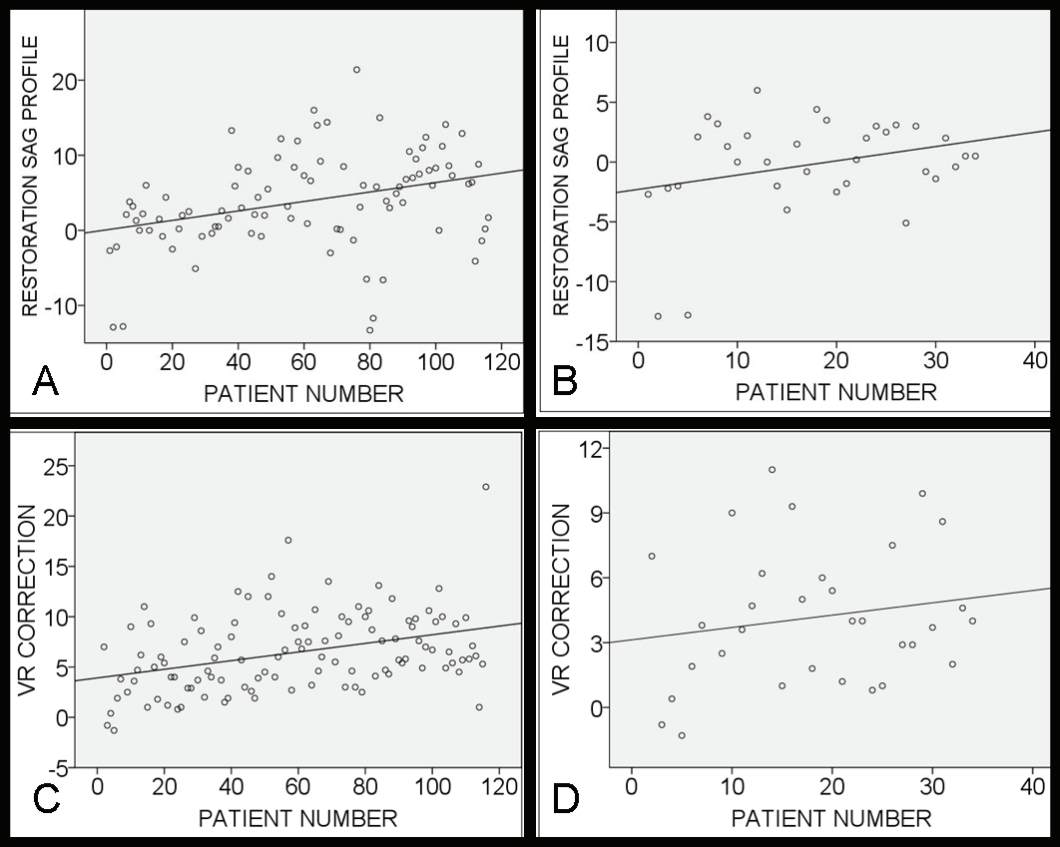
**The impact of rod stiffness on the restoration of the thoracic kyphosis and the correction of the vertebral rotation. **Scatter plot showing the correlation between restoration of the thoracic kyphosis against the patient's number for the whole study population (A), and for the patients operated with smaller rods with a diameter of 5.5 mm (B). Scatter plot showing the correlation between correction of the vertebral rotation against the patient's number for the whole study population (C), and for the patients operated with smaller rods with a diameter of 5.5 mm (D). Footnotes: VR indicates vertebral rotation. Correlation coefficinet = (A) 0.344, (B) 0.232, (C) 0.370, and (D) 0.174.

For the whole study population, there was statistically significant correlation between the order of the operation (patient number) and the restoration of the thoracic kyphosis (r = -0.344, P = 0.001), as well as the correction of vertebral rotation (r = 0.370, P < 0.001) Figure 2A and 2C. There was when using the 5.5 mm rod no significant correlation between the order of the operation and the restoration of the thoracic kyphosis (r = -0.232, P = 0.286) or the vertebral rotation (r = 0.174, P = 0.340), Figure 2B and 2D. Furthermore, there was when using the 5.5 mm rod no mean difference in the restoration of the thoracic kyphosis or the vertebral rotation when the first 17 operated patients were compared with the last 17 patients (P = 0.621, and 0.941, respectively), Table 3.

**Table 3:** The results of the independent sample t-test between first 17 patients and the last 17 patients operated with 5.5 mm rods and one surgeon

Difference between the pre- and the postoperative measures of:	Mean difference	95% CI		P-value
		**Lower bound**	**Upper bound**	
			
Restoration of thoracic kyphosis	1°	-3.1°	5.1°	0.621
Vertebral rotation	0.08°	-2.2°	4.8°	0.941

95% CI indicates 95% confidence interval.

### Age, gender, and curve type

There was no gender difference in the correction of the Cobb angle (p = 0.811) or vertebral rotation (p = 0.225). The age of the patients correlated inversely with correction of the Cobb angle (r = -0.508, p < 0.001) and LEVT (r = -0.375, p < 0.001). There was statistically significant correlation between the correction of the Cobb angle and the correction of vertebral rotation (r = 0.228, p = 0.016) and the LEVT (r = 0.702, p < 0.001) but not with the restoration of the thoracic kyphosis (r = -0.121, p = 0.254). The Cobb angle of the thoracic curves was corrected with 39.4 ± 8.7° compared with 35 ± 9.4° for the thoracolumbar or lumbar curves (P = 0.017). The vertebral rotation of the thoracic curves was corrected with 6.1 ± 4.1° compared with 7.6 ± 2.9° for the thoracolumbar or lumbar curves (P = 0.037).

## Discussion

This study has shown that scoliosis correction using segmental pedicle screw fixation according to Suk, resulted in a deformity correction with reduction of Cobb angle, LEVT, and vertebral rotation by 38.4° (69%), 19° (68%), and 6.5° (37%), respectively, as well as restoration of the thoracic kyphosis by 4° (24%). We also have shown that a significant improvement in correction of axial rotation and restoration of the thoracic sagittal profile was observed between the first period of the study (2005-2006) and the remaining periods of the study (2007-2009). However, the difference between the correction of these deformities among the first 17 and the last 17 patients operated on in the first period of the study was not statistically significant, indicating that the improvement was not the result of the initial learning curve of the surgeon. However, cumulative experience, improvement in surgical techniques including the DVR, and a more frequent use of posterior release as well as a more liberal use of in situ bending of the concave rod prior to the introduction of the stabilising rod on the convexity could possibly might have contributed to successively improved correction in the remaining periods of the study. The use of in situ rod bending is often difficult with stiffer rods and may induce screw loosening, as the manoeuver means applying compression and distraction forces to the screws in this phase of correction. The major improvement of the deformity correction seemed to coincide with the change to stiffer rods (from rod with 5.5 mm to 6.35 mm diameter). The ideal fixation system in the posterior spinal fixation has been a matter of debate. While some studies showed that stiffness of spine implants may far exceed the requirement for successful fusion and predispose to secondary disc degeneration of the adjacent levels [12], others showed that constructs with larger rod diameter resulted in stiffer fusion masses with no evidence of stress shielding [8]. However, the anchors used in the aforementioned study were not pedicle screws.

Our study showed that larger rod diameter and consequently stiffer rods resulted in improvement of the magnitude of deformity correction in the axial and the sagittal planes. This study has also shown that the deformity correction at the end of the study period was comparable with most of the recent reports in the literature [13-15]. The issue of restoration of the thoracic kyphosis has been discussed in many previous reports [16-19]. A reduction of vertebral rotation of 7.1° (42.5%) and a restoration of the thoracic kyphosis varying from 4° [20] to 7° [10] was reported. In the last report [10], the restoration of the thoracic kyphosis by 7° was achieved by using rigid rods (Cotrel-Dubousset 7.0 mm, stainless).

No study is without limitations. One of the limitation of this study was its retrospective nature. This restrained us to analyze for e.g. the preoperative flexibility of the curves as they were not always done with the fulcrum test. This study, however, constitute a consecutive series of patients with AIS of different Lenke that were operated on at one institution using almost the same surgical technique (performed or supervised by one surgeon, the senior author). As Lenke curve types were evenly distributed in different study periods, we assume that the degree of flexibility was also evenly distributed among these groups. Another limitation was the relatively smaller number of patients operated on with a rod diameter of 5.5 mm (n = 34) compared with those operated on with larger rods (n = 84). As mentioned before, cumulative experience, improvement in surgical techniques including the DVR, and a more frequent use of posterior release as well as a more liberal use of in situ bending of the concave rod prior to the introduction of the stabilizing rod on the convexity could possibly might have contributed to successively improved correction in the later periods of the study. We believe that the findings of this study are of adequate clinical significance that warrants further studies evaluating the impact of different degrees of rod stiffness on deformity correction including a larger number of patients.

## Conclusion

This study showed that larger rod diameter (stiffer rods) had a positive impact on the deformity in the sagittal and the axial planes. Initial learning curve seems in our study to be of minor importance for the outcome of the deformity correction.

## Competing interests

The authors declare that they have no competing interests.

## Authors' contributions

KAK has contributed to conception and design of the study, acquisition of data, analysis and interpretation of data, drafting the manuscript and has given his final approval of the version to be published.

MKK has contributed to interpretation of data, revision of the manuscript critically for important intellectual content, and has given his final approval of the version to be published.

ACO has contributed to conception and design, interpretation of data, revising the manuscript critically for important intellectual content, and has given his final approval of the version to be published.
